# Genome-Wide Identification of Metal Tolerance Protein Genes in *Populus trichocarpa* and Their Roles in Response to Various Heavy Metal Stresses

**DOI:** 10.3390/ijms21051680

**Published:** 2020-02-29

**Authors:** Yongfeng Gao, Fengming Yang, Jikai Liu, Wang Xie, Lin Zhang, Zihao Chen, Zhuoxi Peng, Yongbin Ou, Yinan Yao

**Affiliations:** School of Life Science and Engineering, Southwest University of Science and Technology, Mianyang 621010, China; gaoyongfeng0263@gmail.com (Y.G.); myhousework@outlook.com (F.Y.); kateryan@163.com (J.L.); XieWang0808@163.com (W.X.); Zhanglin2636@163.com (L.Z.); chenzihao0856@163.com (Z.C.); lenovopzx@outlook.com (Z.P.); oyb84@swust.edu.cn (Y.O.)

**Keywords:** heavy metal, metal tolerance protein, *Populus trichocarpa*, evolution, gene expression

## Abstract

Metal tolerance proteins (MTPs) are plant divalent cation transporters that play important roles in plant metal tolerance and homeostasis. Poplar is an ideal candidate for the phytoremediation of heavy metals because of its numerous beneficial attributes. However, the definitive phylogeny and heavy metal transport mechanisms of the *MTP* family in poplar remain unknown. Here, 22 *MTP* genes in *P. trichocarpa* were identified and classified into three major clusters and seven groups according to phylogenetic relationships. An evolutionary analysis suggested that *PtrMTP* genes had undergone gene expansion through tandem or segmental duplication events. Moreover, all PtrMTPs were predicted to localize in the vacuole and/or cell membrane, and contained typical structural features of the MTP family, cation efflux domain. The temporal and spatial expression pattern analysis results indicated the involvement of *PtrMTP* genes in poplar developmental control. Under heavy metal stress, most of *PtrMTP* genes were induced by at least two metal ions in roots, stems or leaves. In addition, PtrMTP8.1, PtrMTP9 and PtrMTP10.4 displayed the ability of Mn transport in yeast cells, and PtrMTP6 could transport Co, Fe and Mn. These findings will provide an important foundation to elucidate the biological functions of *PtrMTP* genes, and especially their role in regulating heavy metal tolerance in poplar.

## 1. Introduction

Heavy metal pollution is becoming a more and more serious problem globally. It poses a grave risk for food and environmental safety, as well as human health [1]. For example, Cd and Ni are carcinogenic, teratogenic and mutagenic, while Cu, Hg and Pb can cause neurological diseases [2]. Some plants have hyperaccumulation and/or hypertolerance characteristics for specific heavy metals, and are excellent candidates for phytoremediation to reduce environmental heavy metal pollutant levels. These plants must possess a distinct system that contributes to the high capacity of heavy metal enrichment and tolerance, including metal uptake, efflux, chelation, translocation, intracellular sequestration and storage; among these, the metal transporters play a crucial role [3,4].

Cation diffusion facilitators (CDFs) are divalent cation (Zn^2+^, Co^2+^, Fe^2+^, Cd^2+^, Ni^2+^ and Mn^2+^) transporters which have important functions in maintenance of metal homeostasis. Since the identification of *Cupriavidus metallidurans* in 1995, increasing numbers of CDF genes have been cloned, functionally investigated and classified into three clusters: Zn-CDF, Fe/Zn-CDF and Mn-CDF [5,6]. Protein sequence analysis revealed three typical structural characteristics of CDFs: approximately six predicted transmembrane domains (TMDs); a modified signature; and a C-terminal cation efflux domain [5,7].

Plant CDF family members are usually named metal tolerance proteins (MTPs), which are further classified into seven groups: group 1, group 5–9 and group 12 [8]. Arabidopsis contains 12 *MTP* genes; two members from group1, AtMTP1 and AtMTP3 are vacuolar membrane-localized Zn transporters that function in Zn homeostasis [9,10,11]. AtMTP5 and AtMTP12 are members of groups 5 and 12, respectively. Fujiwara et al. found that these two proteins could interact each other at Golgi for Zn transport [12]. Moreover, AtMTP8 and AtMTP11 are two functionally characterized Mn-CDFs. AtMTP8 is a vacuolar Mn transporter that determines the tolerance to iron deficiency and the localization of Mn and Fe in seeds [13,14,15]. AtMTP11 localizes in prevacuolar compartments and/or trans-Golgi, and plays a role in Mn transport and tolerance [16,17].

Poplar is a model and economically important woody plant, and is also a potential candidate for contaminated soil phytoremediation due to its characteristics, such as easy propagation, fast growth, extensive root systems, high above-ground biomass and immense industrial value [18]. In contrast, only three genes encoding the MTP protein have been cloned and functionally characterized in poplar to date. PtdMTP1 is a vacuolar localized Zn transporter in the form of a homo-oligomer, and would confer Zn tolerance when overexpressed in Arabidopsis [19]. PtMTP11.1 and PtMTP11.2, which showed a punctate localization pattern in yeast like their homolog AtMTP11, could complement the sensitivity of the *Δpmr* mutant to Mn [17]. In 2010, Migeon et al. identified 72 metal transporters including 19 MTP proteins from the *Populous* genome, and analyzed their phylogenic relationship [20]. However, due to the limit of the integrity of the *Populous* genome sequence, a few of MTP members were not identified at that time, and the expression patterns, especially those in response to heavy metal stresses, and the metal transport features of *PtrMTP* genes, are unknown. In this study, we identified 22 PtMTPs from the *Populus trichocarpa* v3.0 genome sequence, and systematically analyzed their evolution and structural features. Moreover, we also explored the expression profiles of the entire *PtrMTP* family under various heavy metal stresses in different tissues, and investigated the potential metal substrates by using a yeast assay. The results of this study are expected to provide useful information with which to better understand the biological functions of PtMTP proteins, which might shed some light on the molecular mechanisms of heavy metal transport and homeostasis in poplar.

## 2. Results

### 2.1. Identification and Classification of MTP Genes in P. trichocarpa Genome

**Figure 1:**
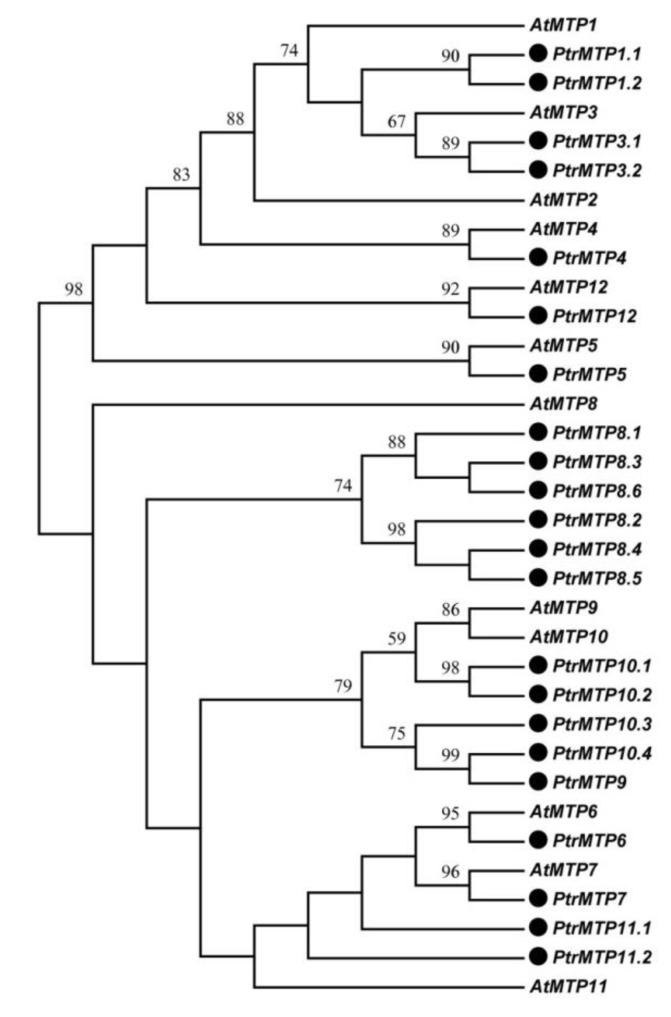
Phylogenetic relationship of MTP proteins in *P. trichocarpae* and *Arabidopsis*. The PtrMTP proteins were named according to the sequence identity and cover values, as well as the orthologous relationship compared with AtMTPs. The black solid circles represent the MTP proteins from *P. trichocarpae*.

By using the sequences of 12 AtMTP proteins as queries, we identified a total of 22 *MTP* genes in *P. trichocarpa* genome, three more than previous results [20]. The 22 PtrMTP proteins were named PtrMTP1.1 to PtrMTP12, according to the phylogenetic relationship, sequence identity and cover values between PtrMTP and AtMTP proteins (Figure 1, Supplementary Table S3, and Table 1). A phylogenetic tree showed that except for AtMTP2, there was at least one homolog of Arabidopsis MTP proteins in *P. trichocarpa* (Figure 1).

**Figure 2:**
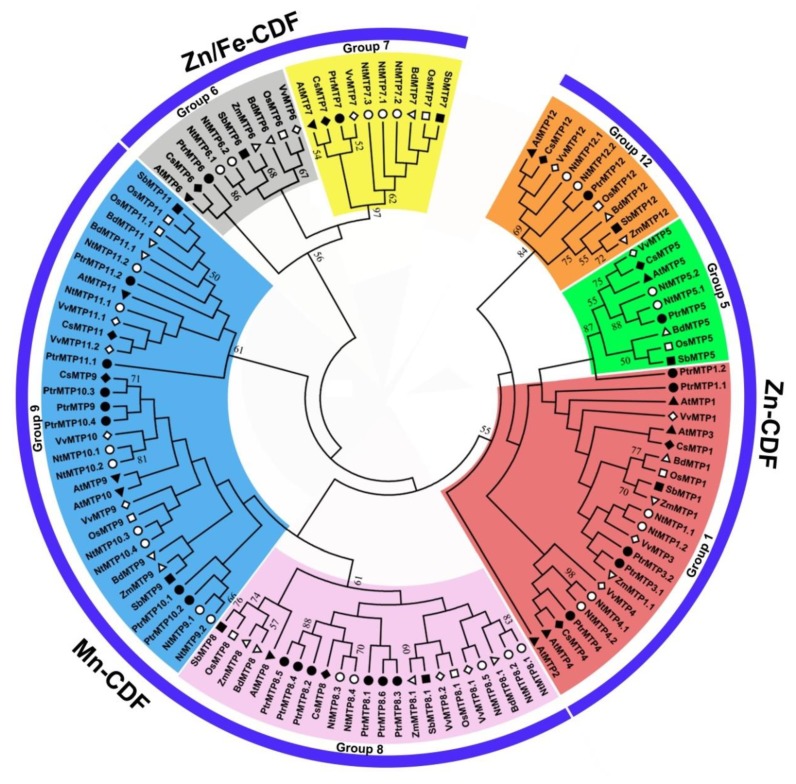
Phylogenetic relationships of MTP proteins in *P. trichocarpae* and other plant species. One hundred and eighteen MTP proteins are clustered into three major substrate-specific groups and seven primary groups, which are highlight in different colors. The different symbols represent the MTP proteins of different species as follows. Solid triangles: *Arabidopsis thaliana*; hollow triangles: *Brachypodium diastychon*; reverse hollow triangles: *Zea mays*; solid diamonds: *Cucumis sativus*; hollow diamonds: *Vitis vinifera*; solid circles: *Populus trichocarpae*; hollow circles: *Nicotiana tabacum*; solid squares: *Sorghum bicolor*; hollow squares: *Oryza sativa*.

To further analyze the phylogenetic relationship of PtrMTP proteins to their counterparts from other plants, a total of 118 MTP sequences from eight plant species were used to construct a phylogentic tree. The 22 PtrMTP proteins could be categorized to seven groups (1, 5, 6, 7, 8, 9 and 12). Among them, group 9 is the largest one, containing 7 PtrMTP members; group 1 includes 5 PtrMTP members, while groups 5, 6, 7 and 12 contain only one PtrMTP each. Amazingly, group 8 contains 5 tandem repeat PtrMTP members from PtrMTP8.2 to PtrMTP8.6 and a single PtrMTP8.1 (Figure 2). Further, the seven PtrMTP groups are classified into Zn-CDFs, Zn/Fe-CDFs and Mn-CDFs clusters.

### 2.2. Structure and Characteristics Analysis of PtrMTP Genes

**Figure 3:**
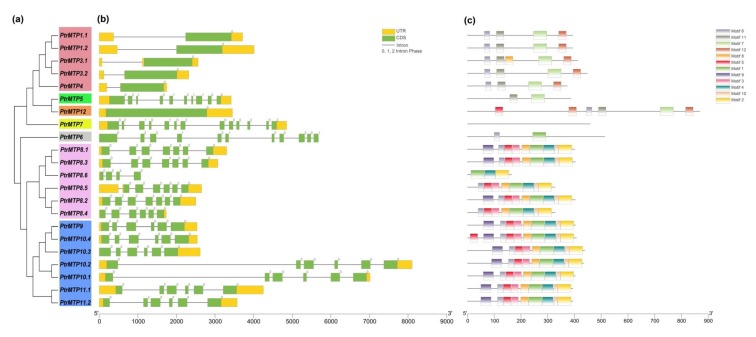
Phylogenetic relationships, gene structure and conserved motifs in *MTP* genes from *P. trichocarpae*. (**a**) A phylogenetic tree was constructed using the MEGA 6.0 software based on the full-length sequences of poplar MTP proteins. Seven primary groups are shown in different colors. (**b**) Exon-intron structure of poplar MTP genes. Yellow boxes indicate untranslated 5′- and 3′-regions (UTR); green boxes indicate exons; gray lines indicate introns. The number indicates the phases of corresponding introns. (**c**) Conserved motifs were identified by MEME and are displayed in different colored boxes.

The genome annotation files of poplar were applied to the TBtools software for an exon-intron organizations analysis of *PtrMTP* genes. As shown in Figure 3a–b, although the number of introns in the *PtrMTP* genes of the three clusters ranged from 0 to 12, most of the related members that clustered closely shared similar introns in terms of number and phase. Of the three clusters, Zn-CDFs contained the smallest number of introns (group 1 contained only one, and group 12 contained none), except for group 5, which harbored 9 introns; Mn-CDFs contained 3–6 introns (group 8 contained 3, 5 or 6, and group 9 all contained 5), whereas Zn/Fe-CDFs contained the largest number of introns (group 6 contained 10, group 7 contained 12) (Figure 3a–b). Additionally, all the *PtrMTP* genes contained phase 0 and phase 2 introns, while only the members of group 5–7 contained phase 1 intron (Figure 3a–b). 

Next, the physicochemical parameters of the 22 PtrMTPs were analyzed further. The length of the coding sequence (CDS) of *PtrMTP* genes ranged from 498 bp (*PtrMTP8.6*) to 2610 bp (*PtrMTP12*), with 165 to 869 amino acids, as well as a relative molecular weight (MW) ranging from 18.375 to 97.498 KDa (Table 1). The total average of hydropathicity (GRAVY) of the PtrMTP proteins ranged from –0.235 (PtrMTP10.1) to 0.329 (PtrMTP4) (Table 1). Moreover, PtrMTP7 has the highest isoelectric point (pI), i.e., 7.24, whereas those of other PtrMTPs were below 7 (Table 1). Furthermore, all PtrMTP proteins were expected to localize in the vacuole, and notably, some (PtrMTP9, PtrMTP10.2, PtrMTP10.3 and PtrMTP10.4) were also localized in the cytoplasm membrane (Table 1). In addition, most PtrMTP proteins contained 4–6 typical TMDs, whereas PtrMTP11.2 had only three, and PtrMTP12 harbored 12; none was found in PtrMTP6 and PtrMTP8.6 proteins (Table 1).

### 2.3. Chromosomal Localization and Gene Duplication Analysis of PtrMTP Genes

**Figure 4:**
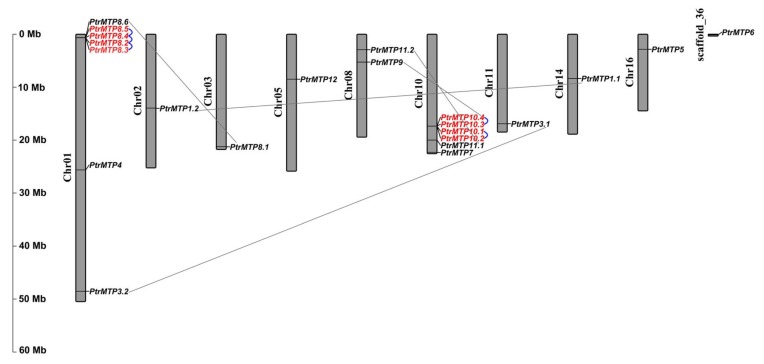
Distribution of the *PtrMTP* genes on *P. trichocarpae* chromosomes. The chromosome number is indicated on the left side of each chromosome, and the size is labeled on the left of the figure. Tandem duplicated genes are outlined with red; tandem and segmental duplicated gene pairs are linked with blue and gray lines, respectively.

To explore the physical locations of the *PtrMTP* genes, genome annotation files were downloaded from the phytozome12 database and analyzed using the TBtools software. The results showed that the 21 out of the 22 *PtrMTP* genes were located on nine poplar chromosomes with an uneven distribution pattern (Figure 4). Most *PtrMTP* genes were assigned to chromosomes 01 and 10, which contained 7 and 6 *PtrMTP* genes, respectively. Interestingly, some *PtrMTP* genes were closely located to one another in a chromosome, such as five *PtrMTP* genes (*PtrMTP8.2-PtrMTP8.5*) on chromosome 01, and four *PtrMTP* genes (*PtrMTP10.1-PtrMTP10.4*) on chromosome 10. *PtrMTP9* and *PtrMTP11.2* were located on chromosome 08, whereas other *PtrMTPs* were separately located on chromosomes 02, 03, 05, 11, 14 and 16, with one *PtrMTP* gene on each chromosome (Figure 4). Nevertheless, the *PtrMTP6* gene located on scaffold_36 could not be mapped onto any chromosome based on the current version of *P. trichocarpa* genome sequence.

The obtained physical locations information of the *PtrMTP* genes prompted us to check the gene duplication events in *PtrMTP* gene family. The results showed that five genes pairs (*PtrMTP8.2/PtrMTP8.3*, *PtrMTP8.2/PtrMTP8.4*, *PtrMTP8.4/PtrMTP8.5*, *PtrMTP10.1/PtrMTP10.2* and *PtrMTP10.3/PtrMTP10.4*) from chromosomes 01 and 10 were identified as tandem duplication events in the *PtrMTP* family (Figure 4). At the same time, five pairs (*PtrMTP1.1/PtrMTP1.2*, *PtrMTP3.1/PtrMTP3.2*, *PtrMTP8.1/PtrMTP8.6*, *PtrMTP9/PtrMTP10.4* and *PtrMTP11.1/PtrMTP11.2*) from six different chromosomes were found as segmental duplication events.

To better understand the selection type of these duplication genes, the ratios of the number of nonsynonymous substitutions per nonsynonymous site (Ka) to the number of synonymous substitutions per synonymous site (Ks) of the 10 gene pairs mentioned above were further calculated. As shown in Table 2, the Ka/Ks ratios of all duplicated pairs of the *PtrMTP* gene were less than 1, which suggested that all these duplication events were under negative selection, based on the summaries from Hurst [21].

### 2.4. Conserved Motif and Domain Architectures Analysis of PtrMTP Proteins

Our study found that PtrMTP proteins contained a total of twelve conserved motifs (Figure 3c), among which only three encode functional domains according to the annotation from the Pfam or InterProScan tools (Figure 3c and Supplementary Table S4). Motifs 1 and 7 encode Cation efflux, while motif 2 encodes ZT_dimer. Motif 6 was widely shared by all PtrMTPs, except for PtrMTP5, PtrMTP7 and PtrMTP8.6 (Figure 3c). Motifs 7, 11 and 12 were mainly distributed in the Zn-CDFs cluster, while motifs 1, 2, 3, 4, 5, 8, 9 and 10 were specifically distributed in the Mn-CDFs cluster. It was also found that the members of the same cluster or group had similar motif types and distributions (Figure 3c). Of the three clusters, Zn/Fe-CDFs contained the smallest number of motifs (group 6 only contained two, and group 7 contained none), Zn-CDFs contained 2–6 motifs (group 1 contained 4 or 5, group 5 contained 2, and group 12 contained 6), whereas Mn-CDFs had the largest number and the most similar types (group 8 contained 8 or 9, and group 9 contained 9 or 10), except for PtrMTP8.6, which contained only three (Figure 3c).

**Figure 5:**
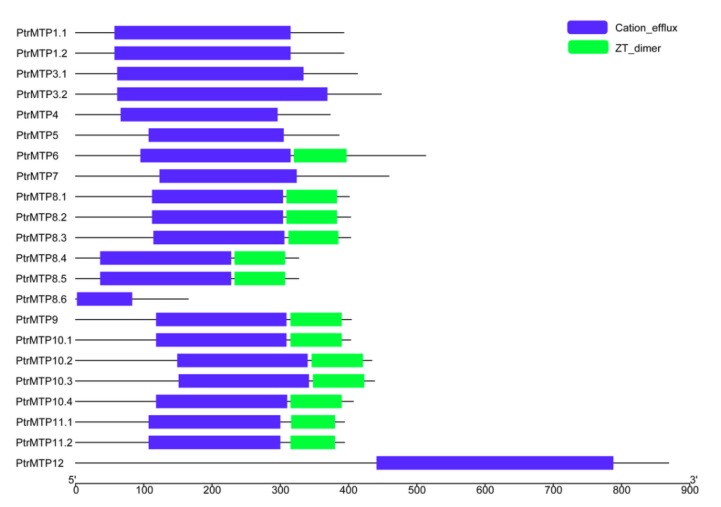
Distributions of the conserved domains in PtrMTP proteins. Blue boxes indicate cation_efflux domains; Green boxes indicate ZT_dimers.

A conserved domain analysis showed that all the PtrMTP proteins contained the cation efflux domain (Figure 5), a typical feature of the MTP protein [5], whereas the members of groups 6, 8 (except for PtrMTP8.6) and 9 possessed a ZT dimer, an important zinc transporter dimerization domain.

### 2.5. Cis-Acting Elements in the Promoter Regions of PtrMTP Genes

A total of 1271 *cis*-acting regulatory elements were identified, which were classified into nine major classes, i.e., 917 elements for gene transcription, 52 elements for abiotic stress, 1 element for biotic stress, 8 elements for tissue expression, 5 elements for secondary metabolism, 80 elements for phytohormonal response, 168 elements for light response, 5 elements for circadian control and 35 elements for site binding (Table 3 and Supplementary Table S5).

Among these, gene transcription elements including 366 CAAT elements and 551 TATA-box elements, which were the most abundance elements, and light responsiveness elements, such as ACE, ATCT-motif, Box 4 and CATT-motif, were commonly present in all *PtrMTPs*. Responsive elements of various phytohormones, such as ABRE, P-box, GARE-motif, TATC-box and SARE, were found in all *PtrMTP* genes, except for the *PtrMTP1.1* and *PtrMTP1.2* genes. Abiotic stress elements including LTR, MBS, TC-rich repeat, WUN-motif, ARE and GC-motif were distributed in the promoters of all *PtrMTP* genes except for *PtrMTP1.1*. In comparison, the AT-rich element involved in biotic stress responsive was detected only in the promoter of the *PtrMTP3.2* gene. Additionally, tissue expression elements including CAT-box and GCN4_motif were present in promoters of *PtrMTP1.*2, *PtrMTP3.2*, *PtrMTP4*, *PtrMTP6*, *PtrMTP9*, *PtrMTP10.1*, *PtrMTP10.3* and *PtrMTP10.4* genes. Moreover, secondary metabolism elements were only detected in the promoters of *PtrMTP1.1*, *PtrMTP3.2*, *PtrMTP6* and *PtrMTP12*, including MBSI involved in flavonoid metabolism and O2-site zein involved in zein metabolism. Notably, site-binding elements were found in all *PtrMTP* genes, except for *PtrMTP1.*1, *PtrMTP7*, *PtrMTP8.5*, *PtrMTP6*, *PtrMTP10.4* and *PtrMTP12*, whereas circadian control elements were only present in promoters of *PtrMTP8.4*, *PtrMTP8.5* and *PtrMTP8.6* (Table 3 and Supplementary Table S5). These results indicated a diverse and complicated control of *PtrMTP* gene expression at the transcriptional level.

### 2.6. Potential miRNA Target Sites in PtrMTP Genes

To explore the probable regulatory mechanism of *PtrMTP* gene expression at the post-transcriptional level, potential miRNAs target sites were predicted using psRNATarget. Finally, we successfully identified a total of 11 miRNAs that targeted 8 *PtrMTP* genes (Table 4). Among these, *PtrMTP12* comprised target sites for three miRNAs (ptc-miR6427-3p, ptc-miR172b-5p and ptc-miR172g-5p), four *PtrMTP* genes, i.e., *PtrMTP7*, *PtrMTP8.1*, *PtrMTP11.1* and *PtrMTP11.2*, contained target sites for two miRNAs, and the remaining three (*PtrMTP1.1*, *PtrMTP3.1* and *PtrMTP10.3*) possessed target sites for single miRNA. Moreover, most of the identified miRNAs function by cleaving target mRNAs, while ptc-miR6426a, ptc-miR6426b, ptc-miR473b and ptc-miR480 work by translation inhibition. In addition, the value of target accessibility-maximum energy to unpair the target site (UPE) of the miRNA/*PtrMTP* varied from 13.362 (ptc-miR480/*PtrMTP7*) to 19.17 (ptc-miR6466-3p/*PtrMTP10.3*). 

### 2.7. The Temporal and Spatial Expression Patterns of PtrMTP Genes

**Figure 6:**
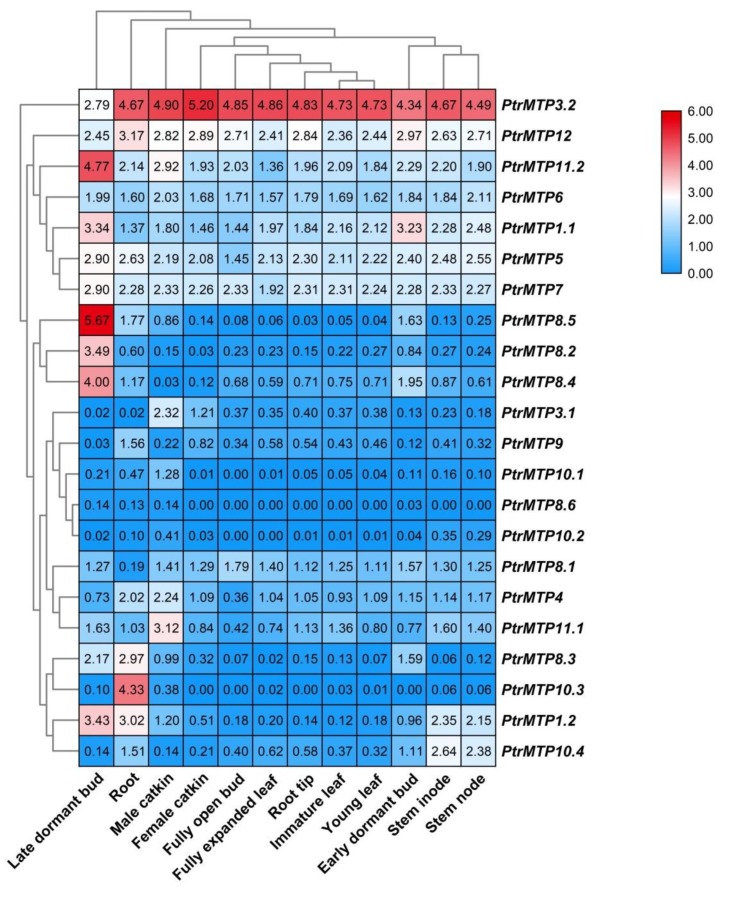
Heatmap analysis of the abundance of *PtrMTP* transcripts in different poplar tissues at different developmental stages. Normalized gene expression (FPKM+1) is expressed in log2 ratio.

The tissue expression patterns of *PtrMTPs* were investigated by using transcriptome data. As shown in Figure 6, all 22 *PtrMTP* genes were expressed in the 12 tested tissues (log2(FPKM+1) > 0), except for *PtrMTP8.6* (which had weak expression only in late dormant bud, root and male catkin) and *PtrMTP10.3* (unexpressed in female catkin, fully open bud, root tip and early dormant bud). Among these, seven genes (*PtrMTP3.2*, *PtrMTP12*, *PtrMTP11.2*, *PtrMTP*6, *PtrMTP1.1*, *PtrMTP5* and *PtrMTP7*) showed constitutive expression (log2(FPKM+1) > 1 in all tissues), and *PtrMTP3.2* had the highest expression levels compared with other *PtrMTPs* in all detected tissues, except for in late dormant bud, whereas two genes (*PtrMTP8.6* and *PtrMTP10.2*) exhibited the lowest expression levels in all tissues (0 < log2(FPKM+1) < 1). Moreover, some genes exhibited tissue-specific expression. For instance, four genes (*PtrMTP8.5*, *PtrMTP8.2* and *PtrMTP8.4*) in late dormant bud, three genes (*PtrMTP9* and *PtrMTP10.3*) in root, four genes (*PtrMTP3.1*, *PtrMTP10.1* and *PtrMTP11.1*) in male catkin, and one gene (*PtrMTP10.4*) in stem nodes showed the highest transcript abundances.

### 2.8. Expression Profiles of PtrMTPs under Different Heavy Metal Treatments

To gain more insight into the gene expression regulatory mechanism of *PtrMTPs*, four-week-old tested tube plantlets of *P. trichocarpa* were subjected to seven different metal treatments. The relative expression levels of *PtrMTPs* in roots, stems and leaves were investigated.

**Figure 7:**
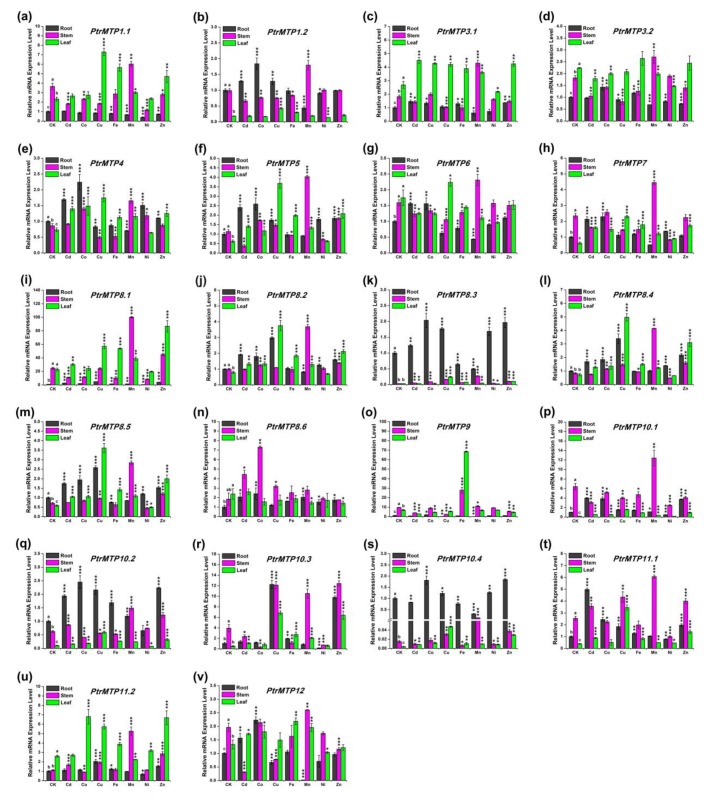
Relative expression levels of *PtrMTP* genes under various metal ion stresses in roots, stems or leaves. Data represent means ± SD of three biological replicates. CK represent control samples. Different letters (a, b and c) indicate significant differences among roots, stems and leaves under normal condition (*n* = 9, *p* < 0.05, Student’s *t*-test). Asterisks indicate significant differences between the treatment samples and the corresponding control samples in roots, stems or leaves. (*n* = 9, *p* < 0.05, Student’s t-test). (**a**–**v**) stands for the *PtrMTP1.1*-*PtrMTP12*, respectively.

Under normal conditions, the expression levels of *PtrMTP4*, *PtrMTP8.3*, *PtrMTP8.4*, *PtrMTP8.5*, *PtrMTP10.2* and *PtrMTP10.4* were higher in roots, whereas those of the *PtrMTP1.1*, *PtrMTP7*, *PtrMTP9*, *PtrMTP10.1*, *PtrMTP10.3*, *PtrMTP11.1* and *PtrMTP12* genes displayed higher expression levels in stems, and *PtrMTP3.1*, *PtrMTP3.2*, *PtrMTP11.2* genes displayed higher expression levels in leaves. However, the *PtrMTP1.2*, *PtrMTP5* and *PtrMTP8.2* genes showed similar expression levels in roots and stems, which were higher than those in the leaves. *PtrMTP6*, *PtrMTP8.1* and *PtrMTP8.6* have similar expression levels in stems and leaves, which were higher than those in roots (Figure 7).

We present an overview of the expression levels of all the *PtrMTP* genes under heavy metal toxicity relative to these under normal conditions in Table 5. In detail, we summarized the *PtrMTP* genes in each tissue with expression changes over four times: In root, Cd enhanced the expression of *PtrMTP11.1*; Cu increased the expression levels of *PtrMTP8.1* and *PtrMTP10.3,* but decreased the expression levels of *PtrMTP9*; Mn repressed the expression levels of *PtrMTP9* and *PtrMTP10.3*; Ni also repressed the expression levels of *PtrMTP10.3*, but Zn enhanced its expression. In stem, Cd repressed the expression levels of *PtrMTP12*; Co increased the expression levels of *PtrMTP8.6* but decreased the expression levels of *PtrMTP10.3*; Cu increased the expression levels of *PtrMTP8.3*; Mn increased the expression levels of *PtrMTP8.1*, *PtrMTP8.3*, *PtrMTP8.4*, *PtrMTP8.5*, *PtrMTP10.4* and *PtrMTP11.2*; Ni repressed the expression levels of *PtrMTP10.3*. In leaf, Cu enhanced the expression of *PtrMTP5*, *PtrMTP8.2*, *PtrMTP8.3*, *PtrMTP8.4*, *PtrMTP8.5*, *PtrMTP10.1*, *PtrMTP10.2*, *PtrMTP10.3*, *PtrMTP10.4*, and *PtrMTP11.1*; Fe increased the expression levels of *PtrMTP9*, *PtrMTP10.1*, and *PtrMTP10.3*; Mn increased the expression levels of *PtrMTP10.3*; Zn increased the expression levels of *PtrMTP8.4*, *PtrMTP10.1*, *PtrMTP10.3*, and *PtrMTP10.4* (Figure 7 and Table 5). However, the expression levels of the *PtrMTP3.1*, *PtrMTP3.2* and *PtrMTP6* genes nearly did not change in each tissue under heavy metal toxicity (Figure 7 and Table 5).

### 2.9. Effect of PtrMTP Genes on Yeast Growth

**Figure 8:**
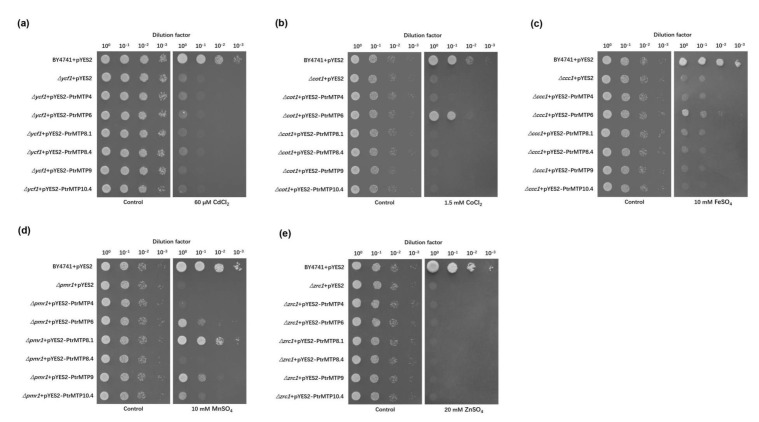
Complementation of yeast mutants on solid medium containing heavy metals. *S. cerevisiae* wild-type strain BY4741 was transformed with the empty vector pYES2, and mutants strains were transformed with the empty vector pYES2 or with the vectors carrying the *PtrMTP* gene, respectively. Yeast cultures were adjusted to OD_600_ = 0.2, and 2 μL of serial dilutions (10-fold, from left to right in each panel) were spotted on SD-Ura/Gal medium supplemented with 60 μM CdCl_2_ (**a**), 1 mM CoCl_2_ (**b**), 10 mM FeSO_4_ (**c**), 10 mM MnSO_4_ (**d**), or 20 mM ZnSO_4_ (**e**) or on the SD-Ura/Glu medium (control) without supplementation. The plates were incubated for 2–4 days at 30 °C. The images are representative for three independent experiments.

According to the expression analysis results and the categories of *PtrMTP* genes, we selected six representative *PtrMTP* genes (*PtrMTP4*, *PtrMTP6*, *PtrMTP8.1*, *PtrMTP8.4*, *PtrMTP9*, and *PtrMTP10.4*) as the objects for a yeast metal sensitivity testing assay. These genes were expressed in the parental strain BY4741 and five yeast mutants that are highly sensitive to Cd (*Δycf1*), Co (*Δcot1*), Fe (*Δccc1*), Mn (*Δpmr1*) and Zn (*Δzrc1*), respectively. As shown in Figure 8, the expression of *PtrMTP6* could rescue the sensitivities of *Δcot1*, *Δccc1* and *Δpmr1* to Co, Fe and Mn, respectively. Moreover, the expressions of *PtrMTP8.1*, *PtrMTP9* and *PtrMTP10.4* alleviated the sensitivity of *Δpmr1* to Mn. However, the expression of *PtrMTP4* and *PtrMTP8.4* could not alter any sensitive phenotypes of the mutants tested. These results suggested that PtrMTP8.1, PtrMTP9 and PtrMTP10.4 could transport Mn^2+^, while PtrMTP6 could transport Mn^2+^, Co^2+^ and Fe^2+^ in yeast cells.

## 3. Discussion

### 3.1. Evolution and Differentiation of PtrMTP Genes as well as Their Proteins Architectures

Here, a total of 22 *PtrMTPs* were identified and named by bioinformatics methods. Compared with that of previous report, three new MTP genesn were found in *P. trichocarpa* [20]. The number of MTPs in *P. trichocarpa* was only second to *N. tabacum* among the plant species in which the *MTPs* have been identified [5,8,22,23,24,25,26,27,28]. However, despite the large number of *PtrMTPs*, the homolog of *AtMTP2* in *P. trichocarpa* was not detected. These results indicated that gene expansion and/or gene loss may have occurred in the history of *PtrMTP* gene family evolution. This hypothesis was later supported by the gene duplication analysis, which revealed that ten duplication events existed in the *PtrMTP* gene family, among which five were segmental and another five were tandem duplications (Figure 4, Table 2). Gene duplication in the genome may lead to the production of new genes with novel functions [29]. Thus, it would be of great interest to investigate the relationship between MTP gene family differentiation and heavy metal tolerance in different plant species in future studies.

As the most typical structural features of MTP proteins, the cation efflux domain and the modified signature sequence were detected in all of the PtrMTPs, although some other motifs/domains were not present in certain PtrMTP members. Interestingly, PtrMTP6 and PtrMTP8.6 did not possess any TMDs (Table 1), a common structure of membrane proteins [30], which suggested that these two proteins might play novel roles, i.e., other than transporters. In addition, it is notable that unlike other PtrMTP members, PtrMTP12 had the largest protein size (869 amino acids), MW (97.50 kD) and TMD number (12 TMDs) (Table 1). This result was consistent with the characteristics of other plants MTP12 [23,25,26], indicating the distinctive biological functions and evolutionary processes of MTP12. Moreover, ZT_dimer has been recognized as the dimerization region of metal ion transporters, through which the homodimers or heterodimers of MTPs can form [31]. In this study, the ZT_dimer was detected in members of groups 6, 8 (except for PtrMTP8.6) and 9, but the question of whether this domain is correlated with their functions requires further investigation (Figure 5). 

### 3.2. Regulation of PtrMTP Gene Expression in P. trichocarpae 

Gene expression control happens at two different levels: one is transcriptional regulation, and the other is post-transcriptional regulation. For the former, *cis*-acting regulatory elements (CRE) play essential roles by interacting with RNA polymerase and specific transcription factors. In this study, the CAAT-box and TATA-box, which are involved in regulating the expression frequency and initiation of transcription respectively [32], were detected in the upstream region of *PtrMTP* genes at a high frequency (Table 3 and Supplementary Table S5). In addition, light-responsive elements, phytohormonal responsive elements and abiotic stress and site-binding elements were also widely distributed in most of *PtrMTP* genes (Table 3 and Supplementary Table S5), implying that *PtrMTP* genes could be transcriptionally regulated by multiple stimuli. 

Previous studies have shown that miRNAs play versatile roles in plant growth and development control and stresses responses [33]. Most of the 11 miRNAs identified in this study perform their functions by cleaving target mRNA, like ptc-miR2111a/b, ptc-miR172b-5p, ptc-miR172g-5p, ptc-miR6427-3p, ptc-miR6464 and ptc-miR6466-3p, while others, e.g., ptc-miR6426a, ptc-miR6426b, ptc-miR473b and ptc-miR480, carry out their functions by translation inhibition. Most of these miroRNAs have been demonstrated to play important roles against environmental stress responses. miR473 was reported only in tree species, and participates in the response to *mycorrhizal* symbiosis and drought in *Poncirus trifoliate* and *populus*, respectively [34,35,36]. miR2111 could be induced by Pi starvation in *Arabidopsis*, but it could also fulfill shoot-to-root translocation to control rhizobial infection in legume roots [37,38]. Moreover, the miR6426 are involved in the response to nutrient deficiencies and contribute to Mg-deficiency tolerance in *Citrus sinensis* [39]. miR172b is a key controller of the autotrophic development transition in *Arabidopsis* [40]. These findings imply the possible involvement of *PtrMTP* in abiotic and biotic stress response through post-transcriptional regulation mediated by miRNAs.

### 3.3. The Diverse Expression Patterns of PtrMTP Genes

Of 22 *PtrMTPs*, 15 exhibited organ/tissue specificity of gene expression during growth and development of poplar, and most *PtrMTP* genes could respond to at least two metal ions in poplar roots, stems or leaves under heavy metal stresses. The abundant *cis*-acting regulatory elements and different expression pattern of the *PtrMTP* gene imply that *PtrMTP* members play an important role in plant development and stress responses. 

Notably, some paralogous genes from the same group, especially some tandem and segmental duplication gene pairs, showed different expression patterns when analyzed by transcriptome data and quantitative RealTime-PCR (qRT-PCR) under various metal ions stresses in different tissues and developmental stages (Figure 6, Figure 7), such as *PtrMPT1.1*/*PtrMPT1.2* and *PtrMPT3.1 /PtrMPT3.2* gene pairs. This may due to the long-term evolution of *PtrMPT* genes, and resulted in the precise and specific regulation mechanism of metal homeostasis in poplar species. Notably, although the majority of tissue expression patterns of *PtrMPT* genes, including groups 1, 3, 4, 5, 6, 7 and 8, were consistent with the result of transcriptome data, some genes belonging to groups 9, 10, 11 and 12 showed some contradictions between the qRT-PCR results and transcriptome data (Figure 7). This inconsistency was also mentioned in our previous literature [26].

Furthermore, the expression of most *PtrMTP* genes could be induced by multiple metals, although some were not putative heavy metal substrates (Figure 7, Table 5). For instance, excess Cu, which is not a potential substrate for the MTP family, could induce the transcription levels of *PtrMTP* genes (Figure 7, Table 5). Similar findings were also made in sweet orange and turnip [24,25]. Other than Mn, the expression of the Mn-CDFs, *PtrMTP9* and *PtrMTP10.3* were sharply upregulated by excess Fe and Zn, respectively (Figure 7, Table 5). On the other hand, MTP genes may not response to their transport substrates. For example, *PtrMTP1.2*, *PtrMTP3.1*, *PtrMTP3.2*, *PtrMTP4* and *PtrMTP12,* which are Zn-CDF genes, underwent no large changes under excess Zn treatment. We speculate that this may be due to the Zn treatment concentrations used in our experiment. In other words, the response of these genes may require higher or lower concentrations of Zn treatment. Certainly, gene response may also happen at the post-transcriptional level, rather than the transcription level. For example, the abundance of the Zn transporter CsMTP1 was increased four-fold in excess Zn treated cucumber roots, whereas the mRNA level of the *CsMTP1* gene was not significantly changed [41]. Other studies have shown that the response of heavy metal transport happened at the post-translational level through changing protein levels, localization and the turnover of transporters [42,43,44,45]. In addition, some *MTPs* even displayed no response to Zn treatment at both the transcriptional and post-transcriptional levels, though it functioned in maintaining intracellular Zn homeostasis by Zn transport, such as AtMTP1 in Arabidopsis [9]. Interestingly, although the accumulation of AtMTP12 was not dependent on Zn concentration, but it could form a functional complex with another MTP, AtMTP5, to transport Zn [12]. These results indicate a complicated and multilayered regulatory mechanism underlying the response of MTPs to heavy metal substrates. Thus, it is necessary to investigate protein level changes under excess heavy metal treatment, and to identify the protein complex of PtrMTPs in future studies.

### 3.4. Some PtrMTPs were Co, Fe and Mn Transporters in Yeast Cells

A yeast–metal sensitivity test assay was performed to clarify the heavy metal substrates of selected PtrMTP transporters. Our results indicated that PtrMTP6, an MTP member with no predictable TMD domain, could transport three different heavy metals: Co, Fe and Mn ions (Figure 8). Similar findings were recently reported by Migocka et al., who found that CsMTP6 could affect iron and manganese homeostasis in cucumber mitochondria [46]. In addition, three PtrMTP members, PtrMTP8.1, PtrMTP9, and PtrMTP10.4, showed specific transport abilities for Mn in yeast cells (Figure 8). These results were in agreement with previous studies. CsMTP8 and OsMTP8.1 are tonoplast-localized Mn transporters which are responsible for Mn tolerance in cucumber and rice, respectively [22,47,48]. Cucumber MTP9 homologue was proved to be a plasma membrane antiporter that functions in Mn^2+^ and Cd^2+^ efflux from root cells [49]. Erbasol et al. found that the Golgi apparatus localized sea beet BmMTP10 was specific to Mn^2+^ transport, with a role in reducing excess cellular Mn^2+^ levels in yeast [50]. Nevertheless, PtrMTP8.4, unlike its paralog PtrMTP8.1, did not show any metal transport abilities in the present study, indicating a functional diversity of PtrMTP within the same group (Figure 8). CsMTP4 from cucumber is the only MTP4 homolog that has been functionally characterized to date. This protein is localized in the vacuolar membranes to sequestrate Zn and Cd into vacuole [42]. However, in our investigation, PtrMTP4 could not transport Zn and Cd as well as other heavy metals in yeast cells (Figure 8). These results suggest that homologs of MTP across plant species may have different biological functions.

## 4. Materials and Methods

### 4.1. Identification and Phylogenetic Analysis of the MTPs in P. Trichocarpa

Twelve *MTP* genes in *A. thaliana* were downloaded from the TAIR database (https://www.arabidopsis.org/), and were used as queries to perform in TBLASTP searches against the *P. trichocarpa* genome [51]. After analysis with InterProScan (http://www.ebi.ac.uk/interpro/search/sequence-search), the remaining 22 nonredundant candidates were recognized as PtrMTP proteins. Sequence similarity analysis was performed at NCBI (https://blast.ncbi.nlm.nih.gov/Blast.cgi) as described previously [26]. For phylogenetic analysis, sequences of MTP proteins from *P. trichocarpa* and other plant species were first aligned by the Clustal X2.1 [52]; then, phylogenetic trees were established with the Maximum Likelihood method by the MEGA6.06 software [53]. Sequences of MTPs from *B. diastychon*, *C. sativus*, *N. tabacum*, *O. sativa*, *Sorghum bicolor*, *V. vinifera*, and *Z. mays* were obtained from the databases, as described by Liu et al. [26].

### 4.2. Analysis of Genomic Structure, Chromosomal Localization, Gene Duplication and Ka/Ks of MTPs in P. trichocarpa

The TBtools software (https://github.com/CJ-Chen/TBtools) was used to determine the exon-intron organization and chromosomal localization of *PtrMTP* genes using the genome annotation files downloaded from phytozome12 database (https://phytozome.jgi.doe.gov/pz/ portal.html) [54]. The gene duplication events of *PtrMTP* genes were determined by Multiple Collinearity Scan toolkit (MCScanX) [55]. DnaSP v6 software was used to compute the *Ka* and *Ks* substitution rates, as described by Rozas et al. [56].

### 4.3. Amino Acid Properties and Structure Characteristics of PtrMTP Proteins

For amino acid properties, the ProParam tool (https://web.expasy.org/protparam/), Plant-mPLoc server (http://www.csbio.sjtu.edu.cn/bioinf/plant-multi/) and the TMHMM Server V.2.0 (http://www.cbs.dtu.dk/services/TMHMM/) were used to predict theMW, pI, GRAVY, subcellular localizations and the putative transmembrane regions of PtrMTP proteins, respectively.

For protein structure characteristics, the MEME program (http://alternate.meme-suite.org/tools/meme) and the Pfam tool (http://pfam.xfam.org/search#tabview=tab1) were used to identify the conserved motifs and domains of PtrMTP proteins, respectively. 

### 4.4. Prediction of Cis-Acting Regulatory Elements and MicroRNA (miRNA) Target Sites of PtrMTP Genes

Promoter sequences located 1.0 kb upstream of *PtrMTP* genes were searched using the phytozome12 database, and related *cis*-acting regulatory elements were analyzed using the PlantCare database (http://bioinformatics.psb.ugent.be/webtools/plantcare/html/). The miRNA target sites of *PtrMTPs* were predicted using a small RNA target analysis server (psRNATarget server: http://plantgrn.noble.org/psRNATarget/).

### 4.5. Tissue Expression Pattern Based on RNA-seq Data

The fragments per kilobase of exon model per million mapped reads (FPKM) of *PtrMTP* genes in 12 different tissues in varying developmental stages were downloaded from the Phytozome12 database. Subsequently, the normalized data (log2(FPKM+1)) were used to estimate the expression levels, and a heatmap was created by TBtools software.

### 4.6. Growth Condictions and Heavy Metal Treatments

Tube plantlets of *P. trichocarpa* were grown in a greenhouse with 16 h/8 h photoperiod (24 °C/18 °C). Thirty-day-old plants were then placed in 1/2 Hoagland solutions (pH 6.0) supplemented with different heavy metal, with concentrations of 0.1 mM CdCl_2_, 0.1 mM CoCl_2_, 0.1 mM CuSO_4_, 0.5 mM FeSO_4_, 1 mM MnSO_4_, 0.1 mM NiSO_4_ and 0.5 mM ZnSO_4_, respectively, and normal 1/2 Hoagland solutions were used as the control (CK). Then, 24 h later, the roots, stems and leaves of tube plantlets were collected, and used as materials for RNA extraction.

### 4.7. RNA Extraction and qRT-PCR Analysis

RNA extraction, cDNA preparation and qRT-PCR were performed as suggested by Liu et al. with minor modifications [26]. Each experiment was performed with three technical replicates. Two house-keeping genes, *UBQ* (GenBank accession LOC7455401) and *EF1α* (GenBank accession LOC18100225), were used as internal reference. The primers used for qRT-PCR are listed in Supplementary Table S1. The qRT-PCR programs were as described by Gao et al. [57]. The relative expression values were calculated using the 2^–ΔΔ Ct^ method [58].

### 4.8. Yeast Transformation and Growth Assay

The full coding regions of six *PtrMTP* genes were amplified from the cDNAs that was reverse-transcribed from the total RNA of plant leaves. The specific primers used for yeast expression vector construction are presented in Supplementary Table S2. The PCR products were then inserted into *Kpn* I + *Xba* I or *Hind* III + *Xba* I sites of the pYES2.0 vector.

In this study, the following wild-type and mutants of yeast (*Saccharomyces cerevisiae*) strains obtained from the Euroscarf (http://www.euroscarf.de/index.php?name=News) were used: Y00000 (BY4741), Y00829 (*Δzrc1*), Y04534 (*Δpmr1*), Y01613 (*Δcot1*), Y04069 (*Δycf1*), and Y04169 (*Δccc1*). Yeast transformations were performed by LiOAc/PEG method [59]. The solid synthetic drop-out (SD) uracil medium with glucose (SD-Ura/Glu) was used for the selection of yeast transformants. Subsequently, overnight yeast cultures were applied in drop assays, which were performed as described by Liu et al. [26].

## 5. Conclusions

In this study, 22 MTP members in *P. trichocarpa* were identified, and a systematic and comprehensive analysis of *PtrMTP* genes was performed. The 22 *PtrMTPs* were divided into three major substrate-specific clusters and seven groups. The *MTP* gene family in poplar underwent expansions in MTP1, MTP3, MTP8, MTP10, and MTP11 compared with those of Arabidopsis, although MTP2 might have been lost to evolutionary history. The structural characteristics of PtrMTP were similar within groups, but were diverse among different groups. The temporal and spatial expression patterns of *PtrMTP* genes were either similar or varied within the same group. In response to different heavy metal stresses, most *PtrMTP* genes were induced by at least two metal ions in roots, stems or leaves. Additionally, PtrMTP8.1, PtrMTP9 and PtrMTP10.4 were found to function as Mn transporters, and PtrMTP6 could transport three different heavy metal ions, i.e., Co, Fe and Mn in yeast cells, indicating that these proteins might play important roles in heavy metal homeostasis, detoxification and tolerance in poplar. These results will provide an important foundation for better understanding the mechanism of heavy metal transport mediated by PtrMTP proteins. In addition, our study will also provide important gene resources for the genetic modification of the heavy metal accumulation abilities of plants which can be widely used in phytoremediation.

## Figures and Tables

**Table 1:** Detail information of 22 *PtrMTP* genes identified in current study.

Gene Name.	Gene ID	Chromosome Location	Strand	CDS (bp)	Protein Size(aa)	MW (KDa)	PI	GRAVY	Sub-Cellular Localization	TMD Number
PtrMTP1.1	Potri.014G106200	Chr14:8357551..8361095	+	1182	393	43.47	5.81	0.074	Vacuole	6/In-In
PtrMTP1.2	Potri.002G180100	Chr02:13987567..13990838	+	1182	393	43.55	5.9	0.07	Vacuole	6/In-In
PtrMTP3.1	Potri.011G150600	Chr11:16906810..16909370	−	1242	413	45.24	6.02	0.064	Vacuole	6/In-In
PtrMTP3.2	Potri.001G450900	Chr01:48519109..48521426	+	1347	448	48.94	5.85	−0.174	Vacuole	6/In-In
PtrMTP4	Potri.001G245800	Chr01:25633268..25635016	+	1122	373	41.43	5.47	0.329	Vacuole	6/Out-Out
PtrMTP5	Potri.016G045200	Chr16:2844025..2847434	−	1161	386	43.24	6	0.173	Vacuole	6/In-In
PtrMTP6	Potri.T034500	scaffold_36:109770..116219	+	1542	513	55.86	6.58	−0.012	Vacuole	0
PtrMTP7	Potri.010G251300	Chr10:22330375..22335229	−	1380	459	50.51	7.24	−0.017	Vacuole	4/In-In
PtrMTP8.1	Potri.003G215600	Chr03:21264228..21267534	+	1206	401	45.08	5.24	0.038	Vacuole	5/In-Out
PtrMTP8.2	Potri.001G010200	Chr01:654690..657190	−	1212	403	45.31	6.04	−0.033	Vacuole	5/In-Out
PtrMTP8.3	Potri.001G010300	Chr01:657997..661070	−	1212	403	45.62	5.2	−0.034	Vacuole	4/Out-Out
PtrMTP8.4	Potri.001G010100	Chr01:651043..652778	−	984	327	36.95	5.51	0.205	Vacuole	5/In-Out
PtrMTP8.5	Potri.001G010000	Chr01:647003..649031	−	984	327	37.11	5.78	0.149	Vacuole	5/In-Out
PtrMTP8.6	Potri.001G009900	Chr01:643504..644577	−	498	165	18.38	5.48	0.048	Vacuole	0
PtrMTP9	Potri.008G083600	Chr08:5257637..5260169	−	1215	404	46.52	6.68	−0.165	Cell membrane/Vacuole	5/In-Out
PtrMTP10.1	Potri.010G172800	Chr10:17367355..17374280	+	1212	403	46.52	6.77	−0.235	Vacuole	6/In-In
PtrMTP10.2	Potri.010G172900	Chr10:17376155..17384476	+	1305	434	50.17	6.8	−0.198	Cell membrane/Vacuole	4/Out-Out
PtrMTP10.3	Potri.010G172700	Chr10:17359356..17361845	+	1317	438	49.89	6.24	−0.064	Cell membrane/Vacuole	5/In-Out
PtrMTP10.4	Potri.010G172600	Chr10:17355982..17358522	+	1224	407	46.73	6.88	−0.076	Cell membrane/Vacuole	5/In-Out
PtrMTP11.1	Potri.010G211300	Chr10:19986602..19990567	+	1185	394	44.87	5.05	−0.053	Vacuole	4/Out-Out
PtrMTP11.2	Potri.008G049600	Chr08:2924867..2928439	−	1185	394	44.74	4.88	−0.055	Vacuole	3/Out-In
PtrMTP12	Potri.005G110300	Chr05:8489679..8492954	−	2610	869	97.5	6.95	−0.026	Vacuole	12/In-In

**Table 2:** Ka/Ks analysis and duplicated date calculation for *PtrMTP* genes.

Duplicatedpair	Duplicate Type	Ka	Ks	Ka/Ks	Positive Selection
*PtrMTP1.1/PtrMTP1.2*	Segmental	0.0752	0.2006	0.374875	No
*PtrMTP3.1/PtrMTP3.2*	Segmental	0.0591	0.3208	0.184227	No
*PtrMTP8.1/PtrMTP8.6*	Segmental	0.0683	0.2624	0.26029	No
*PtrMTP9/PtrMTP10.4*	Segmental	0.0627	0.2097	0.298999	No
*PtrMTP11.1/PtrMTP11.2*	Segmental	0.0388	0.2849	0.136188	No
*PtrMTP8.2/PtrMTP8.3*	Tandem	0.1652	1.1124	0.148508	No
*PtrMTP8.2/PtrMTP8.4*	Tandem	0.0027	0.0044	0.613636	No
*PtrMTP8.4/PtrMTP8.5*	Tandem	0.016	0.0313	0.511182	No
*PtrMTP10.1/PtrMTP10.2*	Tandem	0.0152	0.0257	0.59144	No
*PtrMTP10.3/PtrMTP10.4*	Tandem	0.1409	0.7069	0.199321	No

Notes: Ka/Ks < 1 means negative selection, Ka/Ks = 1 means neutral selection, and Ka/Ks > 1 means positive selection.

**Table 3:** Summary of the *cis*-acting regulatory elements identified in the promoter regions of *PtrMTP* genes.

Gene Name	Gene Transcription	Abiotic Stress	Biotic Stress	Tissue Expression	Secondary Metabolism	Phytohormonal Responsive	Light Response	Circadian Control	Site-Binding
*PtrMTP1.1*	43	0	0	0	2	0	2	0	0
*PtrMTP1.2*	63	4	0	1	0	0	5	0	1
*PtrMTP3.1*	33	3	0	0	0	1	3	0	1
*PtrMTP3.2*	73	4	1	1	1	1	7	0	3
*PtrMTP4*	41	3	0	1	0	5	12	0	1
*PtrMTP5*	30	1	0	0	0	4	2	0	1
*PtrMTP6*	58	1	0	1	1	3	5	0	1
*PtrMTP7*	34	4	0	0	0	2	4	0	0
*PtrMTP8.1*	57	1	0	0	0	6	12	0	4
*PtrMTP8.2*	20	3	0	0	0	2	5	0	1
*PtrMTP8.3*	32	3	0	0	0	7	13	0	3
*PtrMTP8.4*	20	2	0	0	0	3	7	1	2
*PtrMTP8.5*	39	2	0	0	0	2	9	2	0
*PtrMTP8.6*	20	3	0	0	0	3	6	2	2
*PtrMTP9*	20	1	0	1	0	1	5	0	0
*PtrMTP10.1*	33	2	0	1	0	7	15	0	4
*PtrMTP10.2*	33	2	0	0	0	7	14	0	2
*PtrMTP10.3*	27	1	0	1	0	5	4	0	1
*PtrMTP10.4*	45	1	0	1	0	1	6	0	0
*PtrMTP11.1*	25	5	0	0	0	11	12	0	5
*PtrMTP11.2*	60	1	0	0	0	3	11	0	3
*PtrMTP12*	111	5	0	0	1	6	9	0	0

**Table 4:** The potential miRNA target sites in *PtrMTP* genes.

miRNA Acc.	Target Acc.	Expectation	UPE	miRNA Length	Target Start-End	miRNA Aligned Fragment	Target Aligned Fragment	Inhibition
ptc-miR473b	PtrMTP1.1	2.5	15.471	20	932–951	GCUCUCCCUCAGGGCUUCCA	UUGAAGUCCUGAUGGAGAGC	Cleavage
ptc-miR2111a	PtrMTP11.2	3	16.046	21	550–571	UAAUCUGC-AUCCUGAGGUUUG	GCAACUUUAGGAUUGCAGAUUA	Cleavage
ptc-miR2111a	PtrMTP11.1	3	13.919	21	550–571	UAAUCUGC-AUCCUGAGGUUUG	GCAACUUUAGGAUUGCAGAUUA	Cleavage
ptc-miR2111b	PtrMTP11.2	3	16.046	21	550–571	UAAUCUGC-AUCCUGAGGUUUG	GCAACUUUAGGAUUGCAGAUUA	Cleavage
ptc-miR2111b	PtrMTP11.1	3	13.919	21	550–571	UAAUCUGC-AUCCUGAGGUUUG	GCAACUUUAGGAUUGCAGAUUA	Cleavage
ptc-miR6426a	PtrMTP8.1	3	19.01	21	162–182	GUGGAGACAUGGAAGUGAAGA	UUUUCACUUUAAUGUCUCUAA	Translation
ptc-miR6426b	PtrMTP8.1	3	19.01	21	162–182	GUGGAGACAUGGAAGUGAAGA	UUUUCACUUUAAUGUCUCUAA	Translation
ptc-miR6427-3p	PtrMTP12	3	15.602	21	900–920	GUGGGAAUGAACAUUAUGAGA	AAUUAUACUGUUUAUUCCUGC	Cleavage
ptc-miR172b-5p	PtrMTP12	3.5	16.256	21	1705–1725	GGAGCAUCAUCAAGAUUCACA	GGUGGCUCUGGAUCAUGCUCC	Cleavage
ptc-miR172g-5p	PtrMTP12	3.5	16.256	21	1705–1725	GGAGCAUCAUCAAGAUUCACA	GGUGGCUCUGGAUCAUGCUCC	Cleavage
ptc-miR473b	PtrMTP3.1	3.5	18.76	20	989–1008	GCUCUCCCUCAGGGCUUCCA	UGGAGGUUCUCAUGGAGAGC	Translation
ptc-miR480	PtrMTP7	3.5	13.362	24	1122–1145	ACUACUACAUCAUUGACGUUGAAC	AAUAGAUUUCAAUGGAGUAGUGGU	Translation
ptc-miR6464	PtrMTP7	3.5	14.684	21	344–364	UGAUUGCUUGUUGGAUAUUAU	AACAUAGUCAACGAGCAGUCA	Cleavage
ptc-miR6466-3p	PtrMTP10.3	3.5	19.17	21	1009–1029	UAUCAAUCAUCAAAUGUUCGU	GAGAACGUUUGGUCGUUGAUC	Cleavage

**Table 5:** Overview of *PtrMTP* genes in response to different heavy metal stresses.

Gene Name	In Roots							In Stems							In Leaves						
	Cd	Co	Cu	Fe	Mn	Ni	Zn	Cd	Co	Cu	Fe	Mn	Ni	Zn	Cd	Co	Cu	Fe	Mn	Ni	Zn
*PtrMTP1.1*	No	No	No	No	No	-	No	-	No	No	No	No	-	No	No	No	+	+	No	No	+
*PtrMTP1.2*	No	No	No	No	-	No	No	No	No	No	No	No	No	No	No	No	+	No	No	No	No
*PtrMTP3.1*	No	No	No	No	No	No	No	No	No	No	No	+	No	No	No	No	No	No	No	No	No
*PtrMTP3.2*	No	No	No	No	No	No	No	No	No	-	No	No	No	No	No	No	No	No	No	No	No
*PtrMTP4*	No	+	No	No	No	No	No	No	No	No	No	No	No	No	No	+	+	No	No	No	No
*PtrMTP5*	+	+	No	No	No	No	No	-	No	No	No	+	No	No	+	No	++	+	+	No	+
*PtrMTP6*	No	No	No	No	-	No	No	No	No	No	No	No	No	No	No	No	No	No	No	No	No
*PtrMTP7*	+	+	No	No	No	No	No	No	No	No	No	No	-	No	+	+	+	+	No	No	+
*PtrMTP8.1*	+	No	++	No	No	No	+	-	-	No	-	++	-	No	No	No	+	+	No	No	+
*PtrMTP8.2*	No	No	+	No	No	No	No	No	No	No	No	+	No	No	No	No	++	+	No	No	+
*PtrMTP8.3*	No	+	No	No	-	No	No	No	+	++	No	+++	No	+	No	No	++	+	No	No	+
*PtrMTP8.4*	No	No	+	No	No	No	+	No	No	No	No	++	No	No	No	No	++	+	No	No	++
*PtrMTP8.5*	No	No	+	No	No	No	No	No	No	No	No	++	No	No	No	No	++	+	No	No	+
*PtrMTP8.6*	+	+	No	No	+	No	No	+	++	No	No	No	No	No	No	No	No	No	No	No	No
*PtrMTP9*	No	No	--	No	---	No	No	-	No	-	+	No	No	No	-	No	No	+++	No	No	No
*PtrMTP10.1*	+	+	No	No	No	-	+	-	No	No	No	No	-	No	+	+	+++	++	+	No	++
*PtrMTP10.2*	No	+	+	No	No	No	+	No	No	No	No	+	No	No	No	No	++	+	+	No	+
*PtrMTP10.3*	No	No	+++	No	No	--	+++	No	---	+	-	+	--	+	+	No	+++	++	++	No	+++
*PtrMTP10.4*	No	No	No	No	-	No	No	No	No	+	-	+++	No	+	+	+	+++	+	+	+	+++
*PtrMTP11.1*	++	+	No	No	No	No	No	No	No	No	No	+	-	No	+	No	+++	+	No	No	+
*PtrMTP11.2*	No	No	+	No	No	No	No	No	No	No	No	++	No	+	No	+	+	No	No	No	+
*PtrMTP12*	No	+	No	No	----	No	No	--	No	-	No	No	No	No	No	No	No	No	No	No	No

Notes: “+” and “-” indicate 2 < change fold < 4; “+ +” and “- -” indicate 4 < change fold < 8; “+ + +” and “- - -” indicate 8 < change fold < 16; “- - - -” indicates 16 < change fold. “No” indicates that the transcript underwent no change (change fold<2).

## Supplementary Materials

Supplementary materials can be found at https://www.mdpi.com/1422-0067/21/5/1680/s1.

## Abbreviations

MTP	Metal tolerance protein
CDFs	Cation diffusion facilitators
TMDs	Transmembrane domains
MW	Molecular weight
UTR	Untranslated regions
CDS	Coding sequence
pI	Isoelectric point
GRAVY	Grand average of hydropathicity
Chr	Chromosomes
*Ka*	The number of nonsynonymous substitutions per nonsynonymous site
*Ks*	The number of synonymous substitutions per synonymous site
FPKM	Fragments Per Kilobase of exon model per Million mapped reads
qRT-PCR	quantitative RealTime-PCR
SD	Synthetic drop-out
