# Supplementary material for: Genome-Wide Identification of Metal Tolerance Protein Genes in Populus trichocarpa and Their Roles in Response to Various Heavy Metal Stresses
Source: Int J Mol Sci. 2020 Feb 29;21(5):1680. doi: 10.3390/ijms21051680 (PMC7084629; doi:10.3390/ijms21051680)
Supplement: Supplementary file 1 [file ijms-21-01680-s001.pdf]

Supplementary Materials

Supplementary Table S1. Primer sequences of *PtrMTP* genes used for qRT-PCR analysis

Gene name	Forward primer (from 5′ to 3′)	Reverse primer (from 5′ to 3′)
PtrMTP1.1	ACGCATGTGAATTGATTGAGAG	GTTCTTTGGCATTCTTGGAGTT
PtrMTP1.2	TTCTTGTTGCTGCATTCGGT	GCATCTTTTGGGTGCTCGT
PtrMTP3.1	AGGCATAAAAGCCAACAGTCTC	CATAAGTTCGGCGTGGAGTC
PtrMTP3.2	TCGTTCTAGGCACAACAATCG	GGCAACCACCTCATCCATC
PtrMTP4	ATGATCATACTCACAACCACGA	TCTTTGCTGGAGTACCTGATAC
PtrMTP5	GTATTCTACTGCTGAATTGGCG	CACAGCTAACGAGAATGACATG
PtrMTP6	GCTTTACTGGGTAACAAAACGAG	CAAGAATAGCCCCTCCAACAC
PtrMTP7	CAAAAAGGTTTGCTTTTTGGGG	GCTCGTTGACTATGTTGATCAC
PtrMTP8.1	GAGAGTTGTGGACCGAGTGGA	GGAAGGATTTTAACGTCGCA
PtrMTP8.2	TTTCGGAGTATGGATGATTGTAAT	GCTCTGTAACAGCCTCTGACC
PtrMTP8.3	GGCATTCGTTCATCTTGATTTT	GAAACCAACGCCAACTCTGAC
PtrMTP8.4	GGGAAATGACAAAGACAATGAGG	AAGAATGCCACCAGCCATAAG
PtrMTP8.5	CCAAAACAACAGGCTTAACAGA	CATCATCCTCATTGTCGTTGTC
PtrMTP8.6	GAAACAGTATTGCCCGTGCT	AGTTCAATGTCAACACGCTCG
PtrMTP9	TTTTGCCTGGAAGTTTAAGCG	ACTGCCAATGATCTGCTCTCAGT
PtrMTP10.1	CCATTACTTTGCGGAGGTTG	TGCAGTGAAAGGTTGGGCT
PtrMTP10.2	AAAAGCCAAACCATTACCACC	TCCTGCCCCTTATCCATTTC
PtrMTP10.3	CAAATCAGGTTGTGGGAAATAA	CAGACAAGTGGAAACAGGAAAG
PtrMTP10.4	ATGAAGTGGAATCATTCGTTG	TTTGGTTTTTTCATAGCGTAAG
PtrMTP11.1	GAGTTTTAGGTCCTGAAGATGATG	ATGGCTAATGAACCACTTCTAACA
PtrMTP11.2	AGGCCATTAGACACATTGATACA	AAGAGATCATATTCACCTACAACCA
PtrMTP12	GTATCTTCAGTCATTCCGTTGC	CAAATCCTTGATTCCGACATCC
PtrEF1a	GGCAAGGAGAAGGTACACAT	CAATCACACGCTTGTCAATA
PtrUBQ	GGCAAGACCATCACTCTCGA	ACCTCAAGGGTGATGGTCT

Supplementary Table S2. Primer sequences of *PtrMTP* genes used for yeast expression plasmids construction

Primer name	Primer sequence (from 5′ to 3′)	Restriction enzyme site
**PtrMTP4-pYES2-F**	GGGGTACCATGGAACATGACGGAGTCTC	*KpnI*
**PtrMTP4-pYES2-R**	GCTCTAGATCTGAAAGCTACTACTCAATTTG	*XbaI*
**PtrMTP6-pYES2-F**	CCCAAGCTTATGGGATTCAAATTCTACAAAAG	*HindIII*
**PtrMTP6-pYES2-R**	GCTCTAGATCCCAACTACTCATTGACAAAC	*XbaI*
**PtrMTP8.1-pYES2-F**	GGGGTACCATGGAGGATATGAATTCTGG	*KpnI*
**PtrMTP8.1-pYES2-R**	GCTCTAGACTAACTGTTGGGTAACCTGC	*XbaI*
**PtrMTP8.4-pYES2-F**	GGGGTACCATGGATACGCTGAAATCCTTT	*KpnI*
**PtrMTP8.4-pYES2-R**	GCTCTAGAAAAGTCAAGGATCATTGCCAG	*XbaI*
**PtrMTP9-pYES2-F**	GGGGTACCATGGCGAGCAGACAGAGC	*KpnI*
**PtrMTP9-pYES2-R**	GCTCTAGAGGCAGTCTCCATTAAAGGATT	*XbaI*
**PtrMTP10.4-pYES2-F**	GGGGTACCATGGTGAGCAAACAGAGC	*KpnI*
**PtrMTP10.4-pYES2-R**	GCTCTAGAACAAGGTAGTCTCCATTAAGG	*XbaI*

Notes: The sequence of restriction enzyme site in the primer was underlined.

Supplementary Table S3. The sequence similarity of MTP proteins between *P. trichocarpae* and *A. thaliana*

Query	AtMTP1	AtMTP2	AtMTP3	AtMTP4	AtMTP5	AtMTP6	AtMTP7	AtMTP8	AtMTP9	AtMTP10	AtMTP11	AtMTP12
**sbjct**	Identity	Identity	Identity	Identity	Identity	Identity	Identity	Identity	Identity	Identity	Identity	Identity
**PtrMTP1.1**	74.38%	64.52%	67.68%	43.21%	26.45%	37.50%	ND	ND	28.12%	28.12%	ND	26.30%
**PtrMTP1.2**	72.46%	62.47%	62.91%	43.58%	28.93%	37.50%	ND	ND	ND	ND	ND	27.73%
**PtrMTP3.1**	65.31%	57.77%	65.48%	40.53%	27.22%	30.19%	25.00%	21.92%	ND	ND	ND	29.94%
**PtrMTP3.2**	63.47%	53.92%	60.63%	37.29%	29.17%	30.19%	20.80%	21.28%	ND	ND	ND	27.85%
**PtrMTP4**	45.65%	46.20%	49.12%	51.72%	20.80%	31.34%	ND	ND	ND	ND	ND	23.76%
**PtrMTP5**	19.82%	20.49%	20.68%	20.73%	73.77%	23.23%	35.00%	21.64%	ND	ND	ND	33.59%
**PtrMTP6**	25.71%	25.44%	27.72%	23.47%	21.43%	63.51%	29.03%	21.40%	21.85%	22.22%	26.27%	28.36%
**PtrMTP7**	21.18%	21.55%	25.37%	ND	35.00%	20.13%	72.26%	ND	ND	21.05%	ND	29.81%
**PtMTP8.1**	ND	ND	ND	ND	ND	23.73%	ND	67.00%	55.19%	49.87%	52.24%	29.31%
**PtrMTP8.2**	ND	ND	ND	ND	ND	20.06%	ND	66.58%	54.88%	54.57%	52.44%	ND
**PtrMTP8.3**	ND	ND	ND	ND	ND	22.04%	ND	66.83%	55.36%	54.88%	54.57%	33.33%
**PtrMTP8.4**	ND	ND	ND	ND	ND	ND	ND	72.29%	59.11%	58.76%	59.30%	ND
**PtrMTP8.5**	ND	ND	ND	ND	22.13%	20.26%	ND	70.18%	58.42%	58.42%	57.89%	ND
**PtrMTP8.6**	ND	ND	ND	ND	ND	20.67%	ND	66.85%	53.49%	52.91%	52.33%	26.51%
**PtrMTP9**	ND	ND	ND	ND	ND	20.56%	31.91%	53.45%	64.58%(cover,92%)	67.92%(cover,86%)	58.20%	29.41%
**PtrMTP10.1**	ND	ND	ND	ND	24.49%	19.38%	ND	55.42%	69.64%	73.57%(e,0;cover,92%)	61.04%	28.57%
**PtrMTP10.2**	ND	ND	ND	ND	40.00%	20.42%	ND	55.12%	69.25%	72.82%(e,0;cover,92%)	60.76%	27.72%
**PtrMTP10.3**	ND	33.33%	ND	ND	ND	21.18%	22.22%	53.22%	66.26%	67.73%(e,0;cover,93%)	60.49%	ND
**PtrMTP10.4**	ND	ND	ND	ND	ND	20.14%	ND	53.29%	65.62%	68.01%(e,0;cover,86%)	58.31%	29.41%
**PtrMTP11.1**	ND	ND	ND	ND	25.00%	19.80%	ND	54.26%	66.67%	60.98%	82.03%	24.75%
**PtrMTP11.2**	ND	ND	ND	ND	23.08%	19.80%	ND	53.78%	60.05%	61.25%	81.52%	24.00%
**PtrMTP12**	27.0%	30.77%	27.97%	25.88%	29.84%	26.67%	ND	ND	28.57%	23.81%	ND	60.02%

Note: In the sequence alignment, each MTP protein of *Arabidopsis thaliana* was used as the query sequence, and all MTP proteins of P. trichocarpae were used as the subject sequence. ND stands for E-vaule ≥ 1.

Supplementary Table S4. The sequences and the Pfam annotations of conserved motifs in PtrMTP proteins

Motif ID	Motif sequence	Length	NSites	E-value	Pfam
**MEME-1**	LWLYCRSFRNEIVRAYAQDHYFDVVTNIVGLIAAVLANKFY WWIDPAGAI	50	13	3.1E-422	Cation_efflux (IPR027469), Cation efflux family
**MEME-2**	YFVEVDIVLPEEMPLKEAHNIGETLQEKJEKLPEVERAFVHL DFECTHKP	50	13	7.8E-390	ZT_dimer (PF16916); Dimerisation domain of Zinc Transporter
**MEME-3**	YPIGKLRMQPVGIVIFASVMATLGLQI	27	12	1.9E-219	No motif was found in Pfam.
**MEME-4**	JALYTITNWSGTVIENVVSLVGRTAPPEFLQKLTYLVWNHH	41	13	2.6E-310	No motif was found in Pfam.
**MEME-5**	ASTLDSLLDLLSGFILWFTHLSMKKPNIY	29	12	1.1E-202	No motif was found in Pfam.
**MEME-6**	ANJVLFAAKVYASIKSGSLAI	21	19	1. 7E-117	No motif was found in Pfam.
**MEME-7**	KKQRNINVQGAYLHVLGDSIQSIGVMIGGAIIWYKPEWKIID LICTLIFS	50	5	5.1E-115	Cation_efflux (PF01545), Cation efflux family
**MEME-8**	JVDKAQPKMSSNQELWLIAIMVSATVVKL	29	12	1.8E-113	No motif was found in Pfam.
**MEME-9**	DLYKTLGRQKKISEYYRKQEELLEGFNEVDALTELGIIP	39	10	7.8E-104	No motif was found in Pfam.
**MEME-10**	IKHIDTVRAYTFGVH	15	12	1.4E-083	No motif was found in Pfam.
**MEME-11**	GWEATPRQSYGYFRIEILGALISIQLIWL	29	5	3.7E-053	No motif was found in Pfam.
**MEME-12**	HELHIWAITVGKILLACHVMIKPDABADM	29	5	3.2E-049	No motif was found in Pfam

Supplementary Table S5. The *cis*-regulatory elements in the promoter regions of *PtrMTP* genes

**Function**	**cis-acting elements**	***PtrMTPs***																					
		1.1	***1.2***	***3.1***	***3.2***	***4***	***5***	***6***	***7***	***8.1***	***8.2***	***8.3***	***8.4***	***8.5***	***8.6***	***9***	***10.1***	***10.2***	***10.3***	***10.4***	***11.1***	***11.2***	***12***
**Gene transcription**						1	1	2	1							1							1
	CAAT	21	26	18	32	5	7	7	5	18	8	16	9	15	13	2	12	12	10	26	8	20	6
						2	1	3	1														9
	TATA-box	22	37	15	41	6	3	1	9	39	12	16	11	24	7	8	21	21	17	19	17	40	5
**Light responsiveness**	ACE										1		1	1									
	ATCT-motif													1						1			
	Box 4	2	2	1	3	4		2		4	3	2	2	3	1	1	4	5		1	3	6	3
	G-Box					4				4		5	1		2	1	6	5	1	1	8	1	2
	GT1-motif		1			2		1	1	2		1		1	2	1				1		2	3
	MRE					2						1		1	1						1	1	1
	AE-box								1										1				
	AT1-motif							2	1			1					1	1		1			
	GATA-motif				2				1								1		1	1			
	Gap-box						1									1							
	I-box						1							2			1						
	TCCC-motif											1										1	
	GA-motif			1	1																		
	box II									1		1											
	chs-CMA1a																1	2					
	TCT-motif		2	1	1					1	1	1	3			1	1	1	1				
**Abiotic stress responsive**	LTR		1								1	1	1	1			1	1	1		1		
	MBS		1				1		1									1			3	1	1
	TC-rich repeat				1	2																	
	WUN-motif				1	1		1						1	1								2
	ARE		1	2	2				3	1	2	2	1		2	1	1			1	1		2
	GC-motif		1	1																			
**Tissue expression**	CAT-box					1		1								1	1		1	1			
	GCN4_motif		1		1																		

Function	cis-acting elements	*PtrMTPs*																					
		1.1	***1.2***	***3.1***	***3.2***	***4***	***5***	***6***	***7***	***8.1***	***8.2***	***8.3***	***8.4***	***8.5***	***8.6***	***9***	***10.1***	***10.2***	***10.3***	***10.4***	***11.1***	***11.2***	***12***
**Phytohormone responsive**	ABRE ABA					5				2		3	1		2		6	5	1		6	2	1
	P-box gibberellin															1			1	1			
	GARE-motif																						
	Gibberellin								1														
	TATC-box gibberellin																		1				
	SARE																						2
	CGTCA-motif MeJA									1									1		1		2
	TGACG-motif MeJA									1									1		1		
	TGA-element auxin										1		1										
	AuxRR-core auxin						1				1		1	1	1							1	1
	ERE			1	1		3	3	1	2		4		1			1	2			3		
**Biotic stress responsive**	AT-rich sequence				1																		
**Secondary metabolism**	MBSI																						1
	O2-site zein	2			1			1															
**Circadian control**	Circadian												1	2	2								
**Site-binding**	Unnamed 1				1	1	1	1		3	1	3	2		2		3	2	1		3	3	
	Box III		1																				
	CCAAT-box				1																1		
	AT-rich element			1	1					1							1				1		
